# Sclerosing Odontogenic Carcinoma With a Prominent Clear Cell Component Mimicking Odontogenic Clear Cell Carcinoma: An Extremely Rare Case With a Fatal Clinical Outcome

**DOI:** 10.7759/cureus.51429

**Published:** 2024-01-01

**Authors:** Fa-Chih Shen, Katsumitsu Shimada, Rita R Roy, Yutaka Kitamura, Hiromasa Hasegawa

**Affiliations:** 1 Department of Dentistry, Cathay General Hospital Sijhih Branch, New Taipei City, TWN; 2 Department of Clinical Pathophysiology, Matsumoto Dental University, Shiojiri, JPN; 3 Department of Physiology, Matsumoto Dental University, Shiojiri, JPN; 4 Division of Oral and Maxillofacial Surgery, Center of Oral and Maxillofacial Surgery and Dental Implant in Shinshu, Obuse City, JPN; 5 Department of Oral and Maxillofacial Surgery, Matsumoto Dental University, Shiojiri, JPN; 6 Department of Laboratory Medicine, Shinshu University Hospital, Matsumoto, JPN

**Keywords:** ewsr1 fish, metastasis, recurrence, vascular invasion, clear cell morphology, sclerosing odontogenic carcinoma

## Abstract

Sclerosing odontogenic carcinoma (SOC) is an exceedingly rare odontogenic carcinoma known for its locally aggressive yet indolent behavior. There have been no reports of metastasis to distant organs, except a single case involving lymph node metastasis. This report details the case of a 49-year-old female who presented with a well-demarcated radiolucent lesion in the mandible, accompanied by root resorption and tooth displacement. Microscopically, the lesion exhibited a distinctive composition, with two distinct components: cords of epithelium embedded within an abundant collagenous stroma and solid nests of clear polygonal cells surrounded by hyalinized stroma. Notably, the tumor exhibited direct invasion into the submental muscles, accompanied by perineural and vascular invasion, as well as cortical bone loss. Additionally, the clear cells contained diastase-sensitive periodic acid-Schiff-positive granules. Immunohistochemically, the tumor cells displayed positivity for cytokeratin 19 and p63 while testing negative for myoepithelial markers. The Ki-67 index was measured at 23%. Importantly, neither*EWSR1 *nor *MAML2* rearrangements were detected through fluorescence in situ hybridization (FISH) analysis. Over several years, this patient experienced three instances of local recurrence; notably, four years after the initial surgery, fludeoxyglucose F18-positron emission tomography (18FDG-PET)/CT scans confirmed the presence of pulmonary metastasis. This case presents an unusual histological variation of SOC, marked by vascular invasion, and is notably the first documented case of a fatal outcome in this context.

## Introduction

Sclerosing odontogenic carcinoma (SOC) is an extremely rare odontogenic malignancy. Only 22 cases were reported in the English-language medical literature from 2008 to 2023 [1-6]. SOC is characterized by thin cords or strands of bland odontogenic epithelium infiltrating the prominent fibrous stroma [7]. Clear cell odontogenic carcinoma (CCOC) demonstrates the proliferation of sheets or nests comprising atypical clear cells with abundant glycogen [8]. In some cases, SOC and CCOC appear similar. A case of CCOC mimicking SOC was confirmed through the identification of a fusion gene involving the Ewing sarcoma RNA-binding protein 1 (*EWSR1*) and activating transcription factor 1 (*ATF1*) genes [9]. Conversely, clear cell differentiation is often observed in SOC, and occasionally, cases without *EWSR1* rearrangement may exhibit CCOC features [2].

Given its rarity, the biological nature of SOC remains controversial. Lim et al. presented SOC as a low-grade carcinoma with no metastatic potential that can be adequately treated with local tumor resection [1]. They doubted the legitimacy of SOC as a malignancy. Fukui et al. reported a case of SOC with solitary lymph node metastasis, suggesting its metastatic potential [5]. To clarify the biological nature of SOC, it will be necessary to accumulate more cases.

Here, we present a case of SOC characterized by a biphasic histological feature, encompassing both SOC and CCOC elements, ultimately resulting in an unfavorable clinical outcome.

## Case presentation

**Figure 1:**
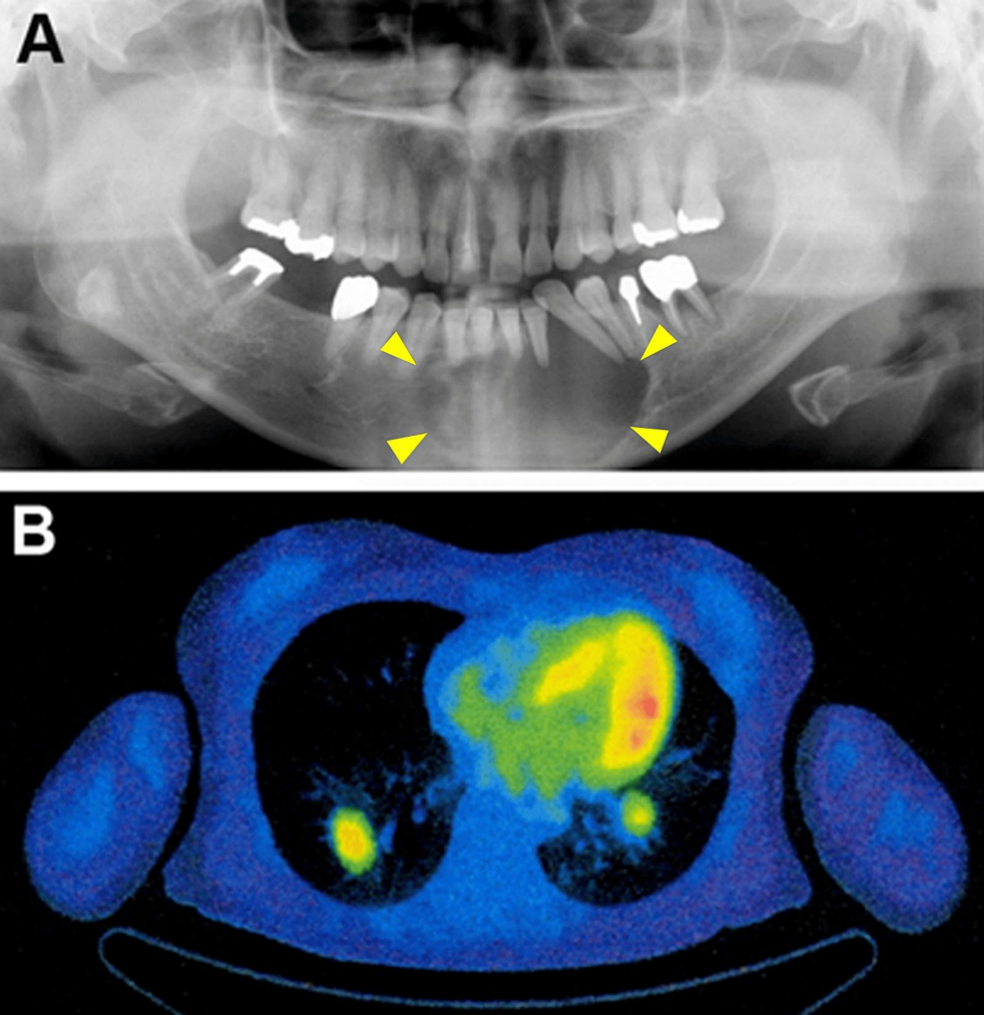
Panoramic X-ray image at the initial administration and 18FDG-PET/CT image four years later (A) The panoramic radiograph at the first visit shows a well-demarcated radiolucent lesion (arrowheads) in the lower anterior mandible; (B) An 18FDG-PET/CT image displays increased uptake in the bilateral lung nodules four years after mandibulectomy 18FDG: fludeoxyglucose F18; PET: positron emission tomography

A 49-year-old female presented at our hospital with a chief complaint of malaligned lower anterior teeth. A preoperative panoramic radiograph revealed a well-demarcated, radiolucent lesion extending from the left second premolar to the right canine regions. This lesion was associated with resorption of the right incisor roots and displacement of the left canine (Figure 1A). A biopsy specimen obtained from the labial side of the mandible with cortical bone loss exhibited a proliferation of solid nests composed of clear cytoplasm. The patient was diagnosed with CCOC and a mandibular segmental resection. The first surgery was performed with a 10 mm safety margin one month after the biopsy. Concurrently, mandibular reconstruction was performed using an autogenous iliac crest bone graft. Due to the discovery of vascular invasion in the surgical specimen, as described below, a comprehensive follow-up, including screening for pulmonary metastasis, was initiated.

As detailed in Table 1, the carcinoma recurred locally three times. During the second recurrence, the removed submental and left submandibular lymph nodes exhibited reactive changes without evidence of metastasis. Four years after the initial surgery, 18F-fluorodeoxyglucose positron emission tomography-computed tomography (18FDG-PET/CT) image revealed increased uptake in bilateral lung nodules (Figure 1B), raising suspicion of pulmonary metastasis. Following a whole-body examination, which detected no malignancies besides the mandibular lesion, pulmonary metastasis was suspected. The patient declined adjuvant therapy based on personal beliefs. She succumbed to respiratory failure eight years after the first surgery, four years after the onset of pulmonary masses.

**Table 1:** Summary of present medical history 18FDG: fludeoxyglucose F18; PET: positron emission tomography

Time-course after the mandibulectomy	Event
Two years	First local recurrence
Three years	Second local recurrence
Four years	Bilateral pulmonary masses suspected with 18FDG-PET/CT
Six years	Third recurrence
Eight years	Died of respiratory failure

**Figure 2:**
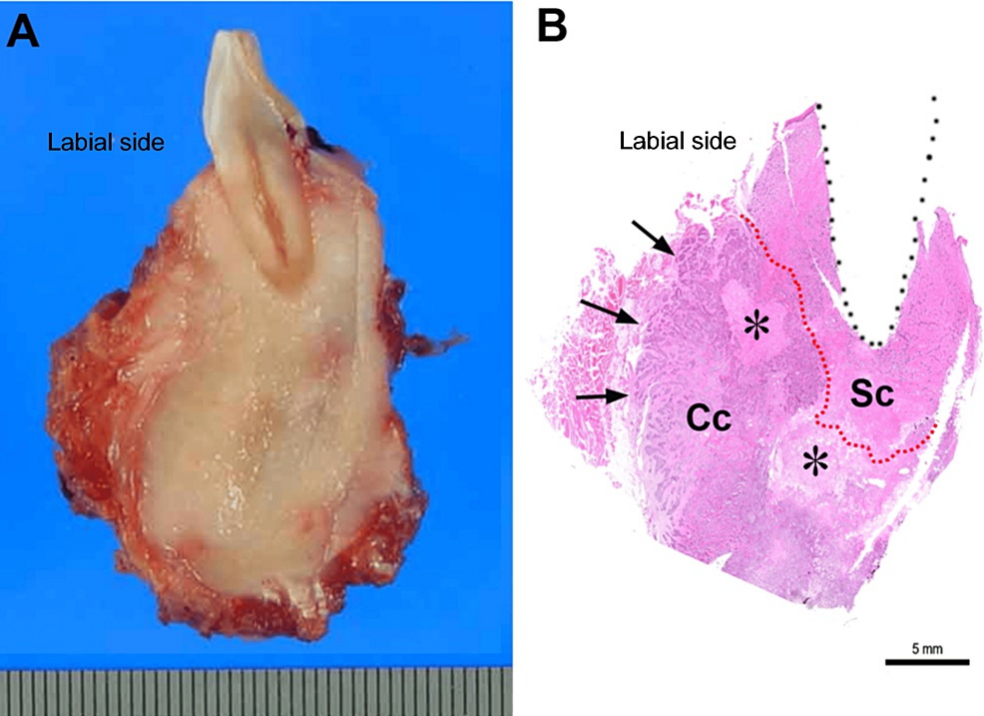
Pathological features The cutting surface of the resected specimen exhibits a relatively well-marginated, light greyish lesion that entirely occupies the anterior alveolar bone, encircling the left first incisor (A). Histologically, muscular invasion (arrows) is evident at the labial side of the mandible. The tumor is divided into two parts, indicated by a red line, with a sclerotic area labeled "Sc" and a higher-cellular area labeled "Cc," featuring ischemic necrosis (asterisks) (B).

Macroscopically, a relatively well-marginated, light-tan tumor extensively occupied the alveolar bone, causing destruction of the labial cortical bone. The tumor measured 35 mm × 30 mm × 25 mm. Microscopically, the resected mandibular bone was entirely replaced by the tumor, with labial cortical bone destruction. The tumor displayed two distinct histological types: a minor sclerotic lesion within a tooth-bearing area and the remaining portion of a highly cellular lesion, which replaced the mandible and invaded the submental muscles. Ischemic necrosis was observed in some areas (Figure 2).

**Figure 3:**
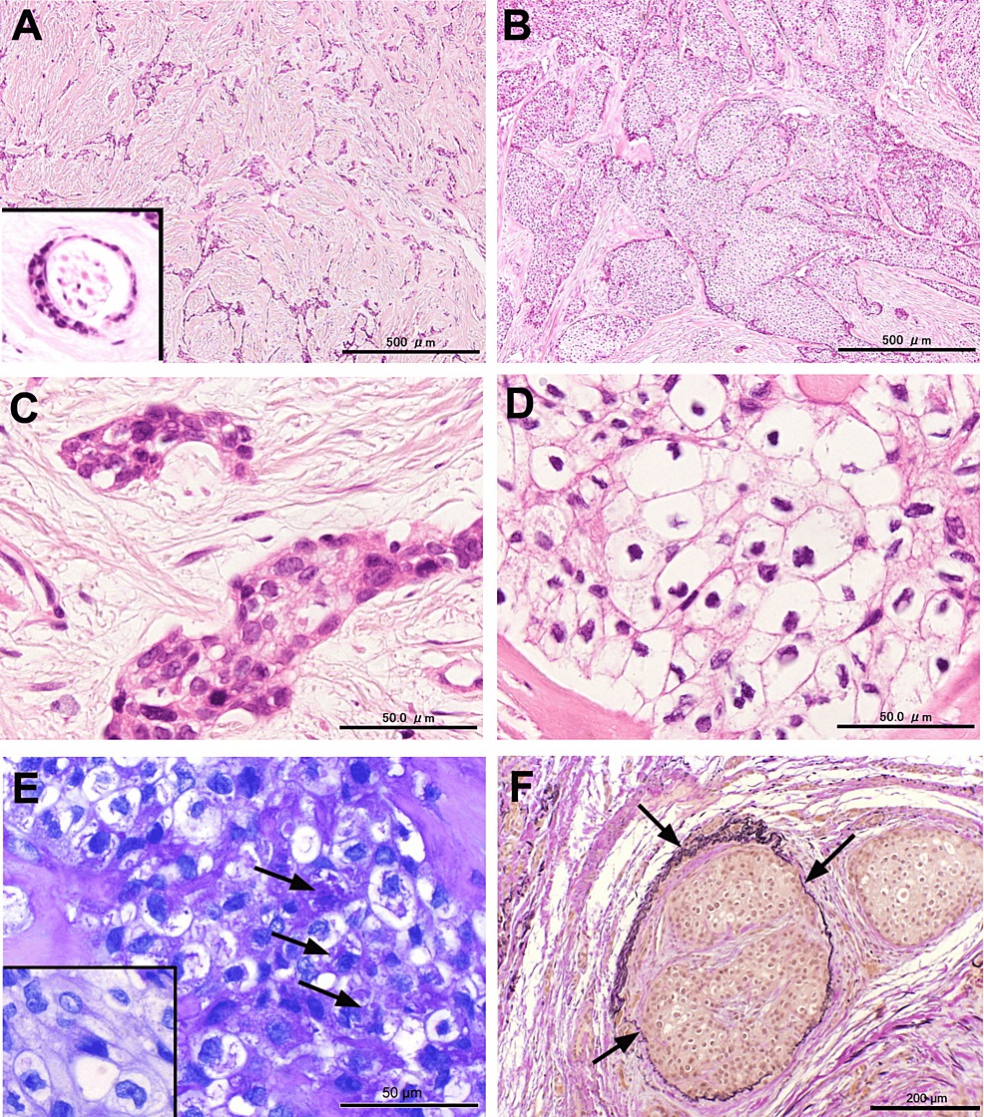
Histopathological findings (A) The tumor comprises a minor area, typifying sclerosing odontogenic carcinoma, with cords of epithelium proliferating within the collagenous stroma. Notably, perineural invasion is observed (inset); (B) The major portion consists of solid nests with hyalinized stroma; (C) The thin cords of nests comprise cuboidal, bland epithelium interspersed with a few atypical cells; (D) Solid nests comprise large polygonal and clear cells with centrally located nuclei exhibiting irregular contours; (E) The clear cytoplasm contains periodic acid–Schiff-positive granules (arrows), which are digested by diastase (inset); (F) Elastin Van Gieson stain highlights tumor nests invading a vascular lumen (arrows)

The minor tumor exhibited typical features of SOC, characterized by the proliferation of cords or strands of epithelium in a prominent collagenous stroma. Tumor nests consisted of cuboidal, bland-looking epithelium with a vacuolated cytoplasm intermixed with a few atypical cells exhibiting hyperchromatic nuclei. On the other hand, the predominant tumor component comprised solid nests with hyalinized stroma. Perineural invasion was evident at the invasive front, invading the muscular tissue. These solid nests comprised large polygonal and clear cells with centrally located atypical nuclei, displaying a hyperchromatic appearance and irregular contours. Peripheral palisading of sliding nests and mitotic figures were absent. The clear cytoplasm contained numerous periodic acid-Schiff (PAS)-positive granules, which were digested by diastase. Elastin Van Gieson Stain revealed significant vascular invasion (Figure 3).

**Figure 4:**
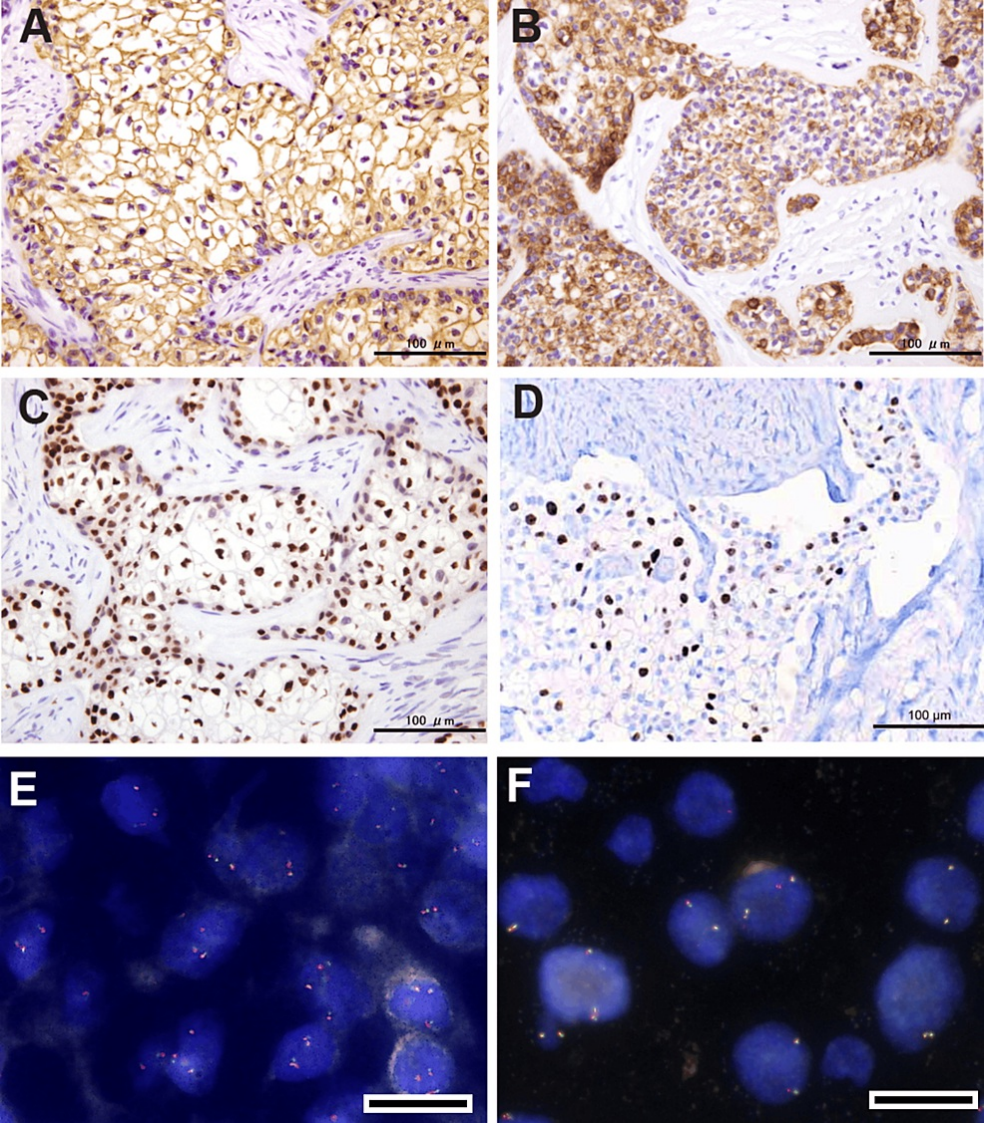
Immunohistochemical and fluorescence in situ hybridization findings All tumor cells demonstrate diffuse positivity for cytokeratin 8 (A), cytokeratin 19 (B), and p63 (C). Ki-67-positive cells are notably prevalent in the hotspots, with an index of 23% (D). FISH analysis using break-apart probes for *EWSR1* (E) and *MAML2* (F) shows no split signals. Bars in (E) and (F) represent 10 μm. FISH: fluorescence in situ hybridization

Mucicarmine staining revealed no mucin deposition or mucus-producing cells in the tested sections. Immunohistochemically, all tumor cells exhibited diffuse positivity for cytokeratin 8, cytokeratin 19, and p63, including the cord-like and solid nests. Ki-67-positive cells were relatively frequent, with an index of 23% in hotspots. Podoplanin (D2-40) immunostaining did not reveal any lymphatic invasion. Other antibodies, such as smooth muscle antibody (SMA), calponin, S100, SOX10, GFAP, and BRAF V600E, showed complete negativity. Fluorescence in situ hybridization (FISH) analyses with break-apart probes for* EWSR1 *and* MAML2* were negative in clear cells, accounting for less than 2%. Consequently, the patient was diagnosed with SOC showing CCOC features due to the absence of *EWSR1 *rearrangement (Figure 4). Immunohistochemical and FISH analyses were conducted on specimens that had not been pretreated or decalcified.

## Discussion

SOC is a primary intraosseous carcinoma with aggressive local infiltration but no metastatic potential [7]. Owing to its rarity and indolent nature, some authors have questioned the legitimacy of SOC as a malignancy [1]. A recent report of recurrence and lymph node metastasis [5] illustrated the true malignant nature of SOC. In addition, the complex histological findings of SOC, which occasionally share histological characteristics with CCOC [5,9], make the diagnosis challenging. The current case may be the first report of SOC with a fatal clinical course and perineural and vascular invasion.

The final diagnosis in this case may be subject to debate, considering the presence of SOC and CCOC features or the possibility of other clear cell tumors. Four potential diagnoses warrant discussion: SOC, CCOC, combined SOC and CCOC, and a collision tumor of SOC and clear cell variant of central mucoepidermoid carcinoma (CMEC). The minor component, localized around the teeth, displayed cord-like epithelial proliferation within a collagenous stroma. The presence of bland and vacuolated cells with minimal atypia is consistent with SOC. Despite histological similarities with benign odontogenic tumors, the presence of perineural invasion underscores its malignant nature and distinguishes it from benign mimics. Considering these morphological and biological characteristics, a diagnosis of SOC can be made for this minor component [7].

In contrast, the major component exhibited infiltrative proliferation of solid nests composed of clear polygonal cells containing glycogen granules, confirmed by digested PAS staining reminiscent of CCOC. Since the tumor lacked calcification, amyloid deposition, peripheral palisading, and BRAF V600E immunoreactivity, other odontogenic tumors with clear cell changes were excluded. Furthermore, the possibility of CMEC was ruled out due to the absence of mucus-producing cells and detectable *MAML2* rearrangement [10]. As a result, differential diagnoses include SOC with CCOC features, CCOC with SOC features, and combined SOC and CCOC.

In addition to the essential diagnostic criteria for CCOC, WHO classification recommends *EWSR1 *translocation as a desirable diagnostic criterion [8]. Because the *EWSR1* split signal could not be identified (accounting for less than 2%), its rearrangement was considered negative. *EWSR1* fusion neoplasms with numerous fusion partners are an extremely broad subset, including soft tissue tumors, specific carcinomas, and a subset of mesotheliomas [11]. In CCOC,* ATF1, CREB1*, and rarely *CREM* are known as *EWSR1* fusion partners [12]. Some Ewing sarcomas with *EWSR1-ERG* fusion are negative for break-apart *EWSR1* FISH. These results emphasize the potential pitfall of relying on the *EWSR1* FISH assay alone for diagnosis [13]. *EWSR1-ERG* fusion demonstrates a complex and unbalanced exchange of chromosomal material between chromosomes 21 and 22 [14]. Namely, the chromosome 22 fragment containing the 5′ portion of* EWSR1* is inverted and inserted into chromosome 21, fusing to the 3′ portion of *ERG* [15]. This phenomenon, or non-*EWSR1* fusion, has not been reported in CCOC. Currently, *EWSR1* FISH is a powerful tool for discriminating CCOC from SOC. The absence of* EWSR*1 rearrangement indicates that it is more likely to be a SOC than a CCOC. 

SOC is typically recognized as a locally aggressive yet slow-growing tumor, generally characterized by an indolent clinical course. Apart from the case reported by Fukui et al. [5], no lymph node or distant organ metastasis instances have been documented in SOC cases. While the bilateral pulmonary masses detected by 18FDG-PET/CT strongly indicated metastatic lesions of SOC, the masses were not histologically examined. A thorough whole-body examination for malignancy did not identify any other primary sites, including the breasts, digestive tract, and female reproductive organs. Synchronous multiple primary lung cancers affect 0.5-2% of lung cancer patients [16], making synchronous bilateral cancers a possibility. Nevertheless, the presence of significant vascular invasion at an early stage strongly suggests the existence of metastatic lesions. The overall clinical course of this case, with the absence of adjuvant therapy, reflects a slow-growing disease. Although SOC is generally considered an indolent carcinoma, it should be acknowledged as a potentially life-threatening tumor.

A mixture of somewhat atypical cells and a Ki-67 index greater than 20% is unusual compared to previously reported cases showing less than 10% [1]. No histological signs have been proposed for predicting the clinical outcomes of patients with SOC or CCOC. The prognosis of CCOC is worse than that of SOC. Approximately 17% of CCOCs metastasize to the regional lymph nodes and rarely to the lungs, pubic bone, brain, vertebrae, and liver [17]. Considering the multiple local recurrences, pulmonary metastasis, and fatal outcomes, the biological behavior of this case was similar to that of CCOC, not SOC. Clear cell nests occupied a greater area, which should be considered when predicting prognosis. However, clear cell differentiation was observed in one-third of previously reported cases, whereas metastasis was not observed in any case [1]. Whether to diagnose this case as SOC or CCOC is still a matter of concern because CCOC with SOC findings may have different fusion genes or abnormalities. Currently, cases should be accumulated for further investigation by focusing on the molecular aspects. It has been suggested that perineural and vascular invasions directly contribute to multiple local recurrences and pulmonary metastases. It is unclear whether atypical features are related to the biological nature of this carcinoma; however, there is no doubt that precise histological examination is necessary to predict the prognosis. 

## Conclusions

The recognition of the life-threatening potential of SOC is crucial, even though it is generally considered an indolent tumor. This case represents an exceptional histological variation of SOC, marked by the presence of vascular invasion; notably, it constitutes the first report of a fatal outcome associated with SOC. These findings underscore the importance of vigilance and comprehensive assessment in cases of SOC, as they may exhibit unexpected aggressive behavior, including the potential for pulmonary metastasis and adverse clinical outcomes.

## References

[1] Lim D, Tan CC, Tilakaratne WM, Goh YC (2022). Sclerosing odontogenic carcinoma - review of all published cases: is it a justifiable addition as a malignancy?. Braz J Otorhinolaryngol.

[2] Seki-Soda M, Sano T, Ogawa I (2020). Two cases of odontogenic carcinoma with sclerosing features in the mandible: diagnostic difficulties in a sclerosing odontogenic carcinoma. J Oral Maxillofac Surg Med Pathol.

[3] Oya K, Tsuji T, Nakatani A (2022). A diagnostic dilemma of sclerosing odontogenic carcinoma: case report. J Oral Maxillofac Surg Med Pathol.

[4] Canterbury CR, Stanbouly D, Trinh K, Clark MS, Philipone E (2023). Sclerosing odontogenic carcinoma: report of a case and review of the literature. Head Neck Pathol.

[5] Fukui R, Yamamoto A, Tsunoda M, Matsumoto K, Namaki S, Asano M (2023). Sclerosing odontogenic carcinoma with local recurrence and lymph node metastasis. Pathology.

[6] Soh HY, Zhang WB, Yu Y, Zhang R, Chen Y, Gao Y, Peng X (2023). Sclerosing odontogenic carcinoma of maxilla: a case report. World J Clin Cases.

[7] Koutlas IG (2022). Sclerosing odontogenic carcinoma. WHO Classification of Tumours: Head and Neck Tumours, 5th edition [AHEAD of PRINT].

[8] Bilodeau EA, Maiorano E, Neville BW (2022). Clear cell odontogenic carcinoma. WHO Classification of Tumours: Head and Neck Tumours, 5th edition [AHEAD of PRINT].

[9] Xie R, Wang W, Thomas AM, Li S, Qin H (2023). Maxillary clear cell odontogenic carcinoma with EWSR1-ATF1 fusion gene mimicking sclerosing odontogenic carcinoma: a case report and literature review. Pathol Res Pract.

[10] Bell D, Holsinger CF, El-Naggar AK (2010). CRTC1/MAML2 fusion transcript in central mucoepidermoid carcinoma of mandible--diagnostic and histogenetic implications. Ann Diagn Pathol.

[11] Jo VY (2020). EWSR1 fusions: Ewing sarcoma and beyond. Cancer Cytopathol.

[12] Breik O, Higginson J, Al-Ajami AK, Mohamed A, Martin T, Amel-Kashipaz R (2021). Clear cell odontogenic carcinoma: first report of novel EWSR1-CREM fusion gene in case of long-term misdiagnosis. Head Neck Pathol.

[13] Chen S, Deniz K, Sung YS, Zhang L, Dry S, Antonescu CR (2016). Ewing sarcoma with ERG gene rearrangements: a molecular study focusing on the prevalence of FUS-ERG and common pitfalls in detecting EWSR1-ERG fusions by FISH. Genes Chromosomes Cancer.

[14] Desmaze C, Brizard F, Turc-Carel C, Melot T, Delattre O, Thomas G, Aurias A (1997). Multiple chromosomal mechanisms generate an EWS/FLI1 or an EWS/ERG fusion gene in Ewing tumors. Cancer Genet Cytogenet.

[15] Kaneko Y, Kobayashi H, Handa M, Satake N, Maseki N (1997). EWS-ERG fusion transcript produced by chromosomal insertion in a Ewing sarcoma. Genes Chromosomes Cancer.

[16] Copur MS, Lackner R, Wedel W, Lintel N, Lintel MS, Gnatra K (2020). Synchronous bilateral lung cancer with discordant histology. Oncology (Williston Park).

[17] Guastaldi FP, Faquin WC, Gootkind F (2019). Clear cell odontogenic carcinoma: a rare jaw tumor. A summary of 107 reported cases. Int J Oral Maxillofac Surg.

